# Visualizing Chain Growth of Polytelluoxane via Polymerization Induced Emission

**DOI:** 10.1002/advs.202304518

**Published:** 2023-09-15

**Authors:** Chengfei Liu, Jinyan Si, Muqing Cao, Peng Zhao, Yiheng Dai, Huaping Xu

**Affiliations:** ^1^ Key Lab of Organic Optoelectronics and Molecular Engineering Department of Chemistry Tsinghua University Beijing 100084 China; ^2^ Tsinghua‐Peking Joint Center for Life Sciences Beijing 100084 China

**Keywords:** oxidative polymerization, polymerization induced emission, polytelluoxane, visual monitoring

## Abstract

Visualizing polymer chain growth is always a hot topic for tailoring structure‐function properties in polymer chemistry. However, current characterization methods are limited in their ability to differentiate the degree of polymerization in real‐time without isolating the samples from the reaction vessel, let alone to detect insoluble polymers. Herein, a reliable relationship is established between polymer chain growth and fluorescence properties through polymerization induced emission. (TPE‐C2)_2_‐Te is used to realize in situ oxidative polymerization, leading to the aggregation of fluorophores. The relationship between polymerization degree of growing polytelluoxane (PTeO) and fluorescence intensity is constructed, enabling real‐time monitoring of the polymerization reaction. More importantly, this novel method can be further applied to the observation of the polymerization process for growing insoluble polymer via surface polymerization. Therefore, the development of visualization technology will open a new avenue for visualizing polymer chain growth in real‐time, regardless of polymer solubility.

## Graphical abstract

Visualizing polymer chain growth is always a hot topic in polymer chemistry. However, current characterization methods are limited in their ability to differentiate the degree of polymerization without isolating the samples from the reaction vessel, let alone to detect insoluble polymers. Instantaneous in‐line analytical methods are proposed to visualize polymer chain growth via oxidative polymerization induced fluorescence emission.

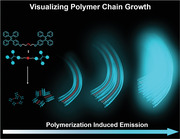

## 1 Introduction

Monitoring and understanding the polymerization process is crucial in polymer science because of its intimate link with structure‐function properties.^[^ ^1^ ^]^ The degree of polymerization is a determining factor in the functional properties of polymers.^[^ ^2^ ^]^ Taking polyethylene as an example, higher degrees of polymerization result in greater hardness and strength.^[^ ^3^ ^]^ Thus, differentiation of polymerization process is a key step in the research of polymer science. To date, diverse monitoring techniques have been employed to monitor the polymerization through molecular weight determination, including gel‐permeation chromatography (GPC),^[^ ^4^ ^]^ NMR spectroscopy,^[^ ^5^ ^]^ MALDI mass spectrometry,^[^ ^6^ ^]^ atomic force microscopy‐based single molecule force spectroscopy,^[^ ^7^ ^]^ and so on. However, most of these characterization techniques require the isolation of products from reaction mixture, leading to the absence of polymerization process characterization in real‐time.^[^ ^8^ ^]^ In addition, the monitoring methods usually require samples to be soluble, which limits the characterization of polymerization degree for insoluble polymers. Therefore, there is an urgent need to develop new characterization methods to differentiate the degree of growing polymers without removing samples from reaction mixture, without the need to consider sample solubility.

Fluorescence‐based techniques can be a powerful and versatile tool for the direct observation of polymerization process in situ, thanks to their high sensitivity, real‐time monitoring, and high‐resolution imaging capability.^[^ ^9^ ^]^ In order to solve the above‐mentioned problem, it is an attractive strategy to establish a relationship between visible fluorescence properties and polymer chain growth by incorporating external fluorophores into polymers. However, the emission of fluorescent materials is always quenched at high concentrations because of the intrinsic aggregation‐caused quenching (ACQ) phenomenon.^[^ ^10^ ^]^ Inspiringly, opposite to the common ACQ effect, materials with the characteristics of aggregation‐induced emission (AIE) can emit intense fluorescence upon aggregate formation,^[^ ^11^ ^]^ which makes them a uniquely promising tool for tracking polymerization process based on their different fluorescence spectra. Alternatively, the issue of polymer degradation is also essential in both academic and industrial fields.^[^ ^12^ ^]^ Unfortunately, since the capabilities of polymerization and depolymerization are opposite to each other, monitoring the depolymerization process still remains a challenge. Given this situation, it is an attractive strategy to establish a relationship between fluorescence properties and polymerization degree to visualize polymerization and depolymerization progress.

**Scheme 1:**
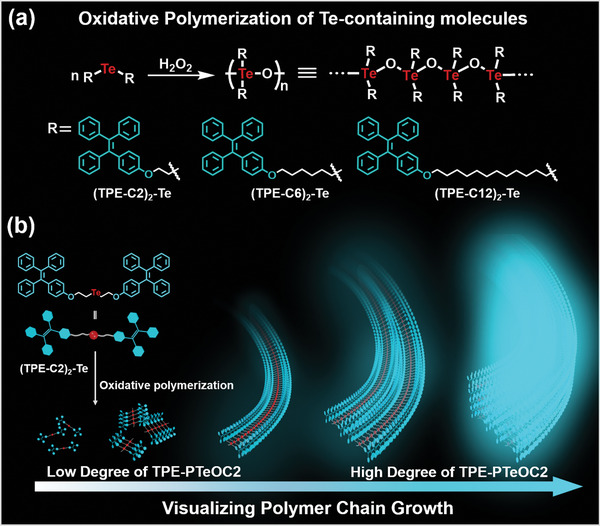
Cartoon representations of visualization of polymer chain growth via oxidative polymerization induced emission. a) Oxidative polymerization of Te‐containing molecules: (TPE‐C2)_2_‐Te, (TPE‐C6)_2_‐Te and (TPE‐C12)_2_‐Te, respectively. b) Schematic illustration of oxidative polymerization process for (TPE‐C2)_2_‐Te and its change of fluorescence color.

In this study, we reported an instantaneous in‐line analytical methods to visualize polymer chain growth of PTeO via oxidative polymerization induced fluorescence emission (**Scheme** **1**). PTeO, as a novel non‐carbon main chain polymer composed of tellurium and oxygen, could be facilely synthesized and further modified into side‐chains with multifunctional properties.^[^ ^13^ ^]^ The acid‐responsive degradation of Te─O backbone enabled the depolymerization of PTeO to be visualized. Owing to the reversible redox property of organotelluride,^[^ ^14^ ^]^ Te‐containing molecules (TPE‐C2)_2_‐Te, (TPE‐C6)_2_‐Te, and (TPE‐C12)_2_‐Te exhibited an ultrasensitive responsiveness to oxidizing agent and conducted in situ oxidative polymerization process (Scheme 1a). As the reaction time increased, the tetraphenylethene (TPE) group in PTeO can emit strong fluorescence in the aggregated state, known as the polymerization induced emission, providing direct and effective information on the entire polymerization process (Scheme 1b). Therefore, the degree of polymerization was correlated with fluorescence color without destroying reaction system. Moreover, surface polymerization could enable the rapid observation of the entire polymerization process of growing polymers, regardless of polymer solubility. Finally, visualizing depolymerization PTeO was also proposed in detail, covering the intriguing and obvious fluorescence changes during the process. Thus, the visualization of polymerization and depolymerization of PTeO can be achieved via polymerization induced emission.

## 2 Results and Discussion

### 2.1 Characterization of Oxidative Polymerization

As for the instant and ultrasensitive responsiveness to H_2_O_2_ of Te‐containing molecules,^[^ ^14^ ^]^ (TPE‐C2)_2_‐Te monomer, containing one tellurium atom and two fluorescent groups (TPE) was first synthesized according to the routes (Scheme S1, Supporting Information). In order to better clarify the relationship between polymerization process and fluorescence properties, we also synthesized two control compounds: (TPE‐C6)_2_‐Te and (TPE‐C12)_2_‐Te monomer with different length of alkyl chain for comparison (Schemes S2 and S3, Supporting Information). The detailed synthetic routes and full characterizations of these Te‐containing molecules are shown in Figures S1–S6 (Supporting Information). PTeO with Te─O bonds as backbones was then formed by an oxidative polymerization between Te‐containing molecules and H_2_O_2_ with a molar ratio of 1: 2 for 12 h at room temperature (Scheme 1a; Figure S7, Supporting Information). Finally, PTeO was fully dissolved in eventual solvents for fluorescence and GPC experiments, constructing a relationship between polymer chain growth and fluorescence properties.

**Figure 1:**
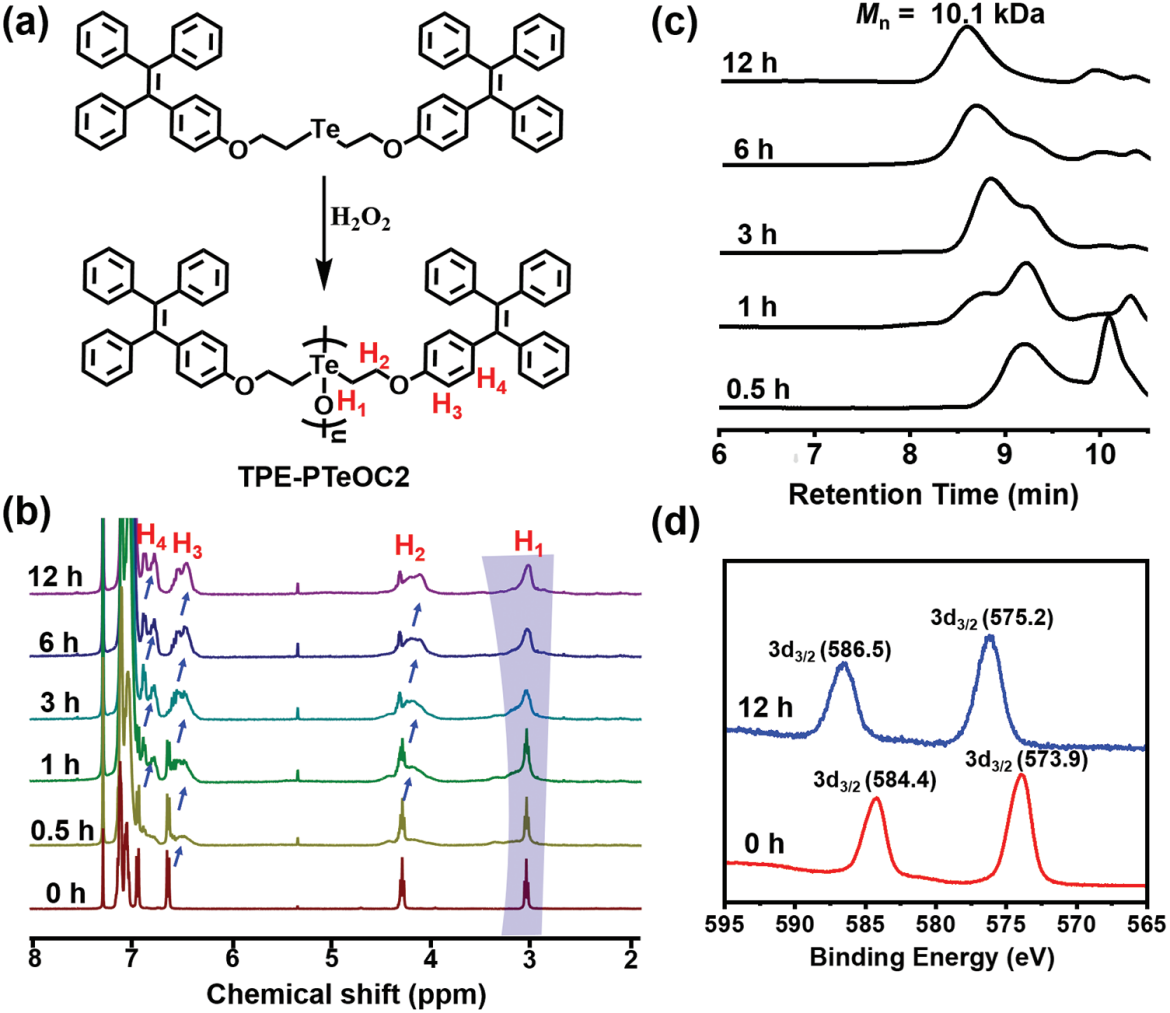
Confirmation of the formation of TPE‐PTeOC2. a) The polymerization process of TPE‐PTeOC2. b) ^1^H NMR characterization of TPE‐PTeOC2 obtained from (TPE‐C2)_2_‐Te incubated with H_2_O_2_ for 12 h. c) GPC analysis of the TPE‐PTeOC2 obtained from (TPE‐C2)_2_‐Te incubated with H_2_O_2_ at different time point. d) Changes in Te 3d binding energy demonstrating the oxidative polymerization.

To investigate the polymerization behaviors of (TPE‐C2)_2_‐Te, in situ ^1^H NMR spectroscopy of (TPE‐C2)_2_‐Te treated with H_2_O_2_ at different time points was first recorded. It is obvious that a significant up field shift of ^1^H NMR resonances for the methylene protons (H_2_) and TPE groups (H_3_ and H_4_) occurred in comparison with the ^1^H NMR spectrum of (TPE‐C2)_2_‐Te monomer (**Figure** **1**b). Upon prolonging the reaction time, all the signals became gradually broader with an obvious vanishing of splitting, providing important evidence for the formation of polymers with a higher degree of repeating units. Meanwhile, the molecular weight of oxidized polymer at different time points was analyzed by GPC. The elution time of products decreased constantly during the time period of 12 h, indicating the formation of (TPE‐C2)_2_‐Te based oxidative polymer (TPE‐PTeO2) with molecular weight of 10.1 kDa (PDI = 1.75) (Figure 1c). Furthermore, the chemical changes for oxidative polymerization were also determined using X‐ray photoelectron spectroscopy (XPS). In Figure 1d, the binding energies of Te 3d at 573.9 and 584.4 eV assigned to Te (+2) disappeared, while the binding energies of Te 3d at 575.2 and 586.5 eV assigned to Te (+4) appeared, indicating an oxidation of Te (+2) to Te (+4). All the results verified the successful formation of TPE‐PTeO2. Meanwhile, (TPE‐C6)_2_‐Te and (TPE‐C12)_2_‐Te monomer could also be oxidized into TPE‐PTeOC6 and TPE‐PTeOC12, respectively, as characterized by ^1^H NMR and GPC experiments. Detailed synthetic procedures and characterization results are shown in Figures S8 and S9 (Supporting Information). All of the results described above confirmed the successful formation of Te─O based oxidative polymers.

### 2.2 Fluorescence Properties of Oxidative Polymer

**Figure 2:**
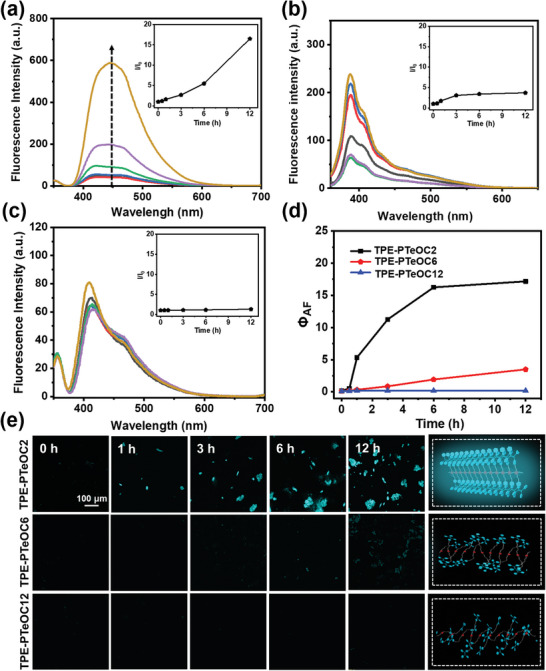
Fluorescence characterization of oxidative polymerization. Fluorescent spectra of (TPE‐C2)_2_‐Te (5 mm) a), (TPE‐C6)_2_‐Te b), and (TPE‐C12)_2_‐Te c) treated with H_2_O_2_ at different time points (*𝜆*_ex_ = 350 nm) in DMF. Inset: the plot of *I*/*I*_0_ at different time points. *I*_0_ = Intensity at 0 h. d) The plot of Φ_AF_ at different time points. e) LSCM images of (TPE‐C2)_2_‐Te, (TPE‐C6)_2_‐Te, and (TPE‐C12)_2_‐Te treated with H_2_O_2_ at different time points and the cartoon representations of its proposed structural arrangement for TPE‐PTeOC2, TPE‐PTeOC6, and TPE‐PTeOC12.

To find out the relationship between the polymerization process and fluorescence properties, the fluorescence spectra of (TPE‐C2)_2_‐Te, (TPE‐C6)_2_‐Te and (TPE‐C12)_2_‐Te solutions under oxidation conditions were recorded, respectively. Prior to that, the fluorescence changes of free TPE incubated with H_2_O_2_ has been evaluated. As shown in Figure S10 (Supporting Information), almost no obvious change in fluorescence intensity was observed as the time continues. Upon the addition of H_2_O_2_ into (TPE‐C2)_2_‐Te solution, fluorescence intensity increased significantly with prolonged polymerization time (**Figure** **2**a). To explore dominative reason for the transition of fluorescence properties, the change of fluorescence intensity for (TPE‐C6)_2_‐Te and (TPE‐C12)_2_‐Te with long alkyl chain was also studied. In Figure 2b, fluorescence intensity increased slightly upon the addition of H_2_O_2_ into (TPE‐C6)_2_‐Te solution, whereas almost no obvious change in fluorescence intensity was observed in (TPE‐C12)_2_‐Te under the same oxidation conditions (Figure 2c). Meanwhile, the corresponding fluorescence quantum yield (Φ_AF_) was also estimated during the process of oxidative polymerization, showing a similar trend to their change in fluorescence intensity (Figure 2d). This phenomenon might be attributed to the polymerization induced tight aggregation among fluorophores, leading to the aggregation of fluorophores emission. The shorter distances between fluorophores, the stronger rigidity of polymer chains, which will increase probability of contacts among fluorophores, leading to the fluorescence emission. Additionally, we can also speculate that fluorescence intensity might correlate with the degree of polymerization for polymer products.

Laser scanning confocal microscopy (LSCM) and dynamic light scattering (DLS) were also used to display the changes in fluorescence color and morphological characteristics for the polymers with polymerization process. The LSCM images of (TPE‐C2)_2_‐Te, (TPE‐C6)_2_‐Te, and (TPE‐C12)_2_‐Te solutions treated with H_2_O_2_ were generated under UV excitation at different polymerization time points, respectively. As Figure 2e and Figure S11 (Supporting Information) showed, only tiny particles were observed at the initial oxidative polymerization time, displaying blue fluorescence. Then, the particles disappeared, accompanied by the generation of the light blue aggregates. As the polymerization went on, fibrous structures formed a thin and compact lamellated structure contributing to more chain‐to‐chain contacts. Meanwhile, the fluorescence intensity increased significantly with prolonged polymerization time. On the contrary, the size of particles and fluorescence color didn't change obviously with reaction time continues in both (TPE‐C6)_2_‐Te and (TPE‐C12)_2_‐Te groups. Figure 2e also showed our proposed mechanistic rationale for the fluorescence intensity changes in cartoon fashion. As shown in Figure 2e, the distinct fluorescence colors of TPE‐PTeOC2 could be interpreted as tight contacts among the chains because of the short distances between fluorophores, causing aggregation of fluorophores.

### 2.3 Relationship between Polymer Chain Growth and Fluorescence Properties

**Figure 3:**
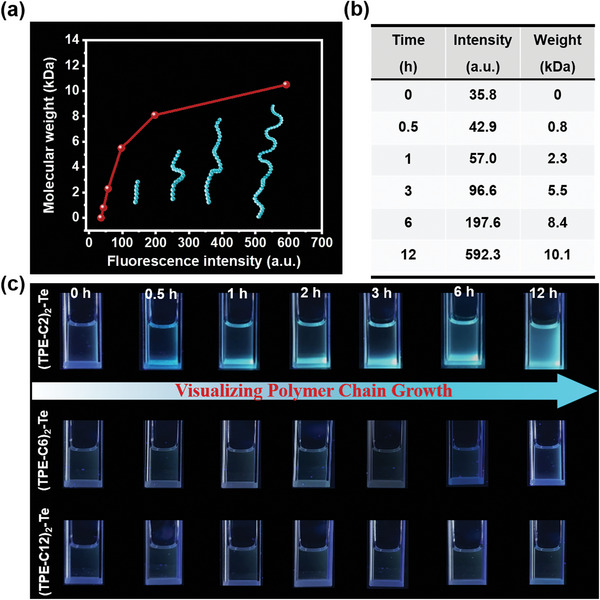
Relationship between polymer chain growth and visible fluorescence properties. a) The plot of corresponding dependent correlation between molecular weight and fluorescence intensity. b) The table of corresponding dependent correlation between molecular weight and fluorescence intensity. c) Photograph of fluorescence changes with the process of (TPE‐C2)_2_‐Te, (TPE‐C6)_2_‐Te, and (TPE‐C12)_2_‐Te based oxidative polymerization under UV irradiation at 365 nm.

The traditional tracking and characterization of polymerization process is often conducted through quenching reaction at precise time point, which is unable to reflect its real‐time information, let alone to monitor an ongoing polymerization continuously. Thus, in situ investigations of polymer chain growth via oxidative polymerization induced emission is believed to be a powerful strategy to overcome the above limitations. In order to obtain the detailed relationship between the fluorescence colors and degree of polymerization process, the quantitative correlation between fluorescence intensity and *M*_n_ should be first constructed. As depicted in **Figure** **3**a,b, we could find that the change in fluorescence intensity is obviously apparent along with the progress of polymerization reaction, suggesting that construction of the useful relationship between the polymer chain growth and fluorescence features is reliable.

Subsequently, the concurrent fluorescence color of the samples was recorded. In Figure 3c, (TPE‐C2)_2_‐Te treated with H_2_O_2_ revealed distinct differences in the fluorescence properties along with polymerization process. At the initial time of reaction, because of the long distances between oligomers, the probability of contacts among TPE was extremely low resulting in the low fluorescence emission. Then blue luminescence was observed and gradually enhanced with longer time, suggesting long chain oxidized polymers were formed causing the aggregation of fluorophores. On the contrary, almost no fluorescence signals were displayed in TPE‐PTeOC6 and TPE‐PTeOC12. The results may be attributed to the fact that the probability of contacts between fluorophores is low, leading to a large separation between the emissive TPE groups. According to the diverse fluorescence colors produced by the polymerization, the corresponding relationship between polymer chain growth and fluorescence colors has been set up. Therefore, this type of correlation may be aided in the future by in‐line visible fluorescence properties, providing new insights into visualization and characterization of polymerization processes in situ.

### 2.4 Visualization of Insoluble Polymer Chain Growth

**Figure 4:**
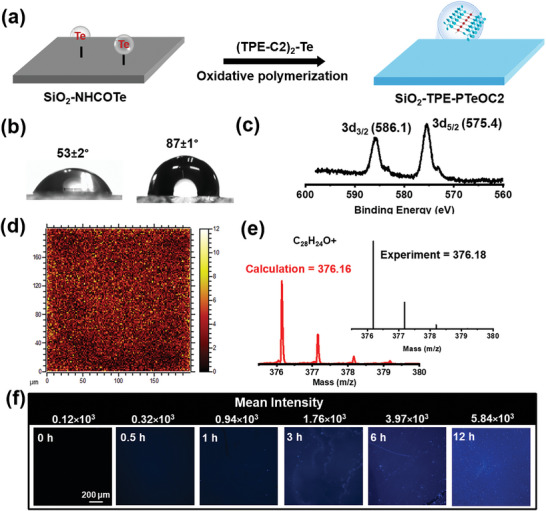
Oxidative polymerization of TPE‐PTeOC2 in surface. a) Schematic illustration of oxidative polymerization in surface on quartz substrates. b) Static water contact angle before (left) and after (right) oxidative polymerization. c) XPS Te 3d spectrum of the SiO_2_‐TPE‐PTeOC2 surface, the binding energy corresponds to Te (+4). d) ToF‐SIMS map of [M+H]^+^(C_28_H_24_O^+^) ions on the SiO_2_‐TPE‐PTeOC2 surface. e) Observed ToF‐SIMS result of the fragment, *M* 376.16. The observed spectra matched well with the calculated one. f) Photograph of fluorescence changes and mean fluorescence intensity with the process of oxidative polymerization under UV irradiation at 420 nm. (Scale bars: 200 µm).

To examine whether or not this type of polymerization induced emission could overcome the insufficient measurement of insoluble polymer chain growth, interfacial polymerization method was developed to track the entire process of growing polymers regardless of their solubility. First, the tellurium‐containing quartz surface denoted as SiO_2_‐NHCOTe was prepared through silylation reaction and amidation reaction (Figures S13 and S14, Supporting Information). Then, we sought to realize the oxidative polymerization reaction on the tellurium containing surface. To demonstrate the mechanism behind surface modification, the whole surface of SiO_2_‐NHCOTe was immersed in a chloroform solution of (TPE‐C2)_2_‐Te, and then placed in H_2_O_2_ solution for oxidative polymerization (**Figure** **4**a). At the end of the reaction, static contact angle increased to 87 ± 1 °C, which may be ascribed to the successful modification of long alkyl chains formed hydrophobic SiO_2_‐TPE‐PTeOC2 surface (Figure 4b). XPS experiments were further conducted to verify the chemical changes in tellurium elements. The binding energies of Te 3d at 586.1 and 575.4 eV were attributed to the Te (4+) of Te‐O (Figure 4c). Finally, the SiO_2_‐TPE‐PTeOC2 surface was also measured by time‐of‐flight secondary ion mass spectrometry (ToF‐SIMS). In Figure 4d,e and Figure S15 (Supporting Information), C_28_H_24_O^+^ and C_56_H_47_O_3_Te^+^ ions from TPE‐PTeOC2 were uniformly distributed on a 200 × 200 µm area. All these results indicated that the SiO_2_‐TPE‐PTeOC2 was successfully formed.

Subsequently, the change of fluorescence colors in the surface of SiO_2_‐TPE‐PTeOC2 was evaluated under the irradiation with 365 nm light at different polymerization time points. After being irradiated by UV light, the surface without immersion of H_2_O_2_ stayed dark since oxidative polymerization cannot be triggered (Figure 4f). On the contrary, the blue luminescence increased significantly along with progress of polymerization reaction, indicating the degree of polymerization gradually increased. The concurrent degree of growing polymers may be roughly estimated by using the previously derived correlation curve of the degree of polymerization and fluorescence intensity. Based on the above results, the visualization of polymerization process for growing insoluble polymer has been well implemented via surface polymerization induced emission.

### 2.5 Visualizing Depolymerization of TPE‐PTeOC2

**Figure 5:**
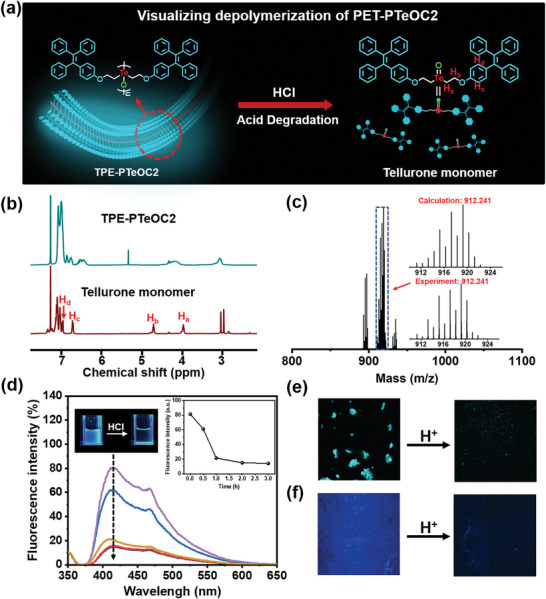
The depolymerization of TPE‐PTeOC2. a) Schematic diagram of depolymerization process of TPE‐PTeOC2 after addition of aqueous HCl. b) ^1^H NMR characterization of TPE‐PTeOC2 incubated with aqueous HCl of 0.5 mm for 6 h. c) ESI‐MS characterization of TPE‐PTeOC2 incubated with aqueous HCl. ESI‐MS showing the peak of tellurone as calculated. d) Fluorescence emission spectra of TPE‐PTeOC2 upon adding aqueous HCl. *λ*_ex_ = 350 nm. Inset: Fluorescent photographs of TPE‐PTeOC2 before and after the addition of aqueous HCl recorded under a handheld UV lamp. e) LSCM images and f) fluorescent photographs of TPE‐PTeOC2 before and after the addition of aqueous HCl.

Since the distinctive fluorescent properties were attributed to the aggregation of TPE groups caused by oxidative polymerization, it was expected that acid depolymerization of TPE‐PTeOC2 may be reflected in a readily discernible change for the fluorescent properties (**Figure** **5**a). To corroborate such a proposition, HCl was added into preformed TPE‐PTeOC2 solutions as for the acid‐responsive degradation of Te─O bonds. In Figure S16 (Supporting Information), the elution time of TPE‐PTeOC2 increased constantly during the time period of 6 h, indicating the successful depolymerization of polymers. Finally, TPE‐PTeOC2 was degraded into tellurone which was characterized by the means of ^1^H NMR (Figure 5b) and ESI‐MS experiments (Figure 5c). The fluorescence characterization was also investigated during the process of depolymerization. As shown in Figure 5d, the fluorescence intensity at 420 nm gradually decreased along with the color changing from blue to dark upon adding aqueous HCl. Additionally, only micro‐sized particles were observed in the corresponding LSCM images (Figure 5e), rather than the cyan lamellated species before the addition of acid solution. Similarly, the photograph of fluorescence also disappeared (Figure 5f). These results verified that emission of fluorophores is quenched due to the separation of TPE groups induced by acid depolymerization of TPE‐PTeOC2. Thus, the visualization of depolymerization process for TPE‐PTeOC2 was realized through use of unique properties of acid depolymerization of PTeO.

## 3 Conclusion

In conclusion, we established a reliable relationship between polymer chain growth and fluorescence properties via (TPE‐C2)_2_‐Te based polymerization induced emission, providing an avenue for visualization of polymerization and depolymerization of PTeO. The evidence for the formation of PTeO was obtained via ^1^H NMR spectroscopy, XPS, and GPC studies. The fluorescence colors of polymerization process were observed. From the spectra, it could be seen that the change in fluorescence colors with the degree of polymerization. This method also charts a path to track the entire polymerization process of insoluble polymers via surface polymerization, which could be further applied to measure some precipitated or high‐molecular weight polymers. Finally, the visualization of depolymerization process was achieved by taking advantage of the fluorophores feature of the degraded PTeO, endowing it with potential industrialization and commercialization value. Overall, we believe that this facile approach will draw significant attention and interest for visualizing polymerization and depolymerization of polymers, and bring inspiration for the rational design and synthesis of polymers with controllable structures and tunable properties.

## Conflict of Interest

The authors declare no conflict of interest.

## Supporting information

Supporting Information

## Data Availability

The data that support the findings of this study are available from the corresponding author upon reasonable request.
