# Supplementary material for: Visualizing Chain Growth of Polytelluoxane via Polymerization Induced Emission
Source: Adv Sci (Weinh). 2023 Sep 15;10(31):2304518. doi: 10.1002/advs.202304518 (PMC10625080; doi:10.1002/advs.202304518)
Supplement: Supplementary file 1 — Supporting Information [file ADVS-10-2304518-s001.pdf]

ADVANCED SCIENCE
Open Access

Supporting Information

for *Adv. Sci.*, DOI 10.1002/advs.202304518

Visualizing Chain Growth of Polytelluoxane via Polymerization Induced Emission

*Chengfei Liu, Jinyan Si, Muqing Cao, Peng Zhao, Yiheng Dai and Huaping Xu**

Supporting Information

Visualizing Polymerization and Depolymerization of Polytelluoxane *via* Polymerization Induced Emission

Chengfei Liu,[a, b]# Jinyan Si,[a]# Muqing Cao,[a] Peng Zhao,[a] Yiheng Dai,[a] and Huaping Xu*[a]

[a] C.F. Liu, J. Y. Si, M. Q. Cao, P. Zhao, Y. H. Dai, Prof. H.P. Xu

Key Lab of Organic Optoelectronics and Molecular Engineering Department of Chemistry, Tsinghua University, Beijing 100084, China

E-mail: xuhuaping@mail.tsinghua.edu.cn

[b] C.F. Liu

Tsinghua-Peking Joint Center for Life Sciences, Beijing 100084, China

Table of Contents

1. Materials and methods

2. Experimental procedures

3. Results and discussion

4. References

1. Materials and methods.

1.1 Materials.

Tellurium powder and hydrogen peroxide 30% aqueous solution were purchased from Aladdin Chemical Company, China. 1,2-dibromoethane, 1,6-dibromohexane and 1,12-dibromododecane were purchased from Tokyo Chemical Industry (TCI). Potassium carbonate (K_2CO_3) and 4-(1,2,2-triphenylvinyl) phenol were purchased from Innochem. 1-(3-dimethylaminopropyl)-3-ethylcarbodiimide hydrochloride (EDC) and 4-dimethylaminopyridine (DMAP) were purchased from Aladdin Chemical Reagent Co. Ltd. 3-aminopropyltriethoxysilane was purchased from J&K Chemical. Other organic solvents, such as dichloromethane (DCM), ethyl acetate, methanol, tetrahydrofuran (THF) used in this work were purchased from Beijing Chemical Reagent Company, China.

1.2 Instrumentation.

^{1}H NMR and ^{13}C NMR spectra were obtained from BRUKER ASCENDTM 400 spectrometer. The GPC measurements were performed by Waters 515 (Milford, MA) (standard: polystyrene, eluent: THF). Electrospray Ionization Mass Spectrometry were acquired with TOF-Q II 10280 (Varian Inc., USA). Ultraviolet visible (UV-vis) spectra were recorded on a UV-2450-visible spectrophotometer (Shimadzu, Japan). The X-ray photoelectron spectroscopy (XPS) was employed by a PHI Quantera scanning X-ray microprobe. The Time of-Flight Secondary Ion Mass Spectrometry (ToF-SIMS) was performed on a ION-TOF GmbH TOF-SIMS.

2. Experimental Procedures.

2.1 Synthesis of $(TPE\text{-}C2)_2$-Te

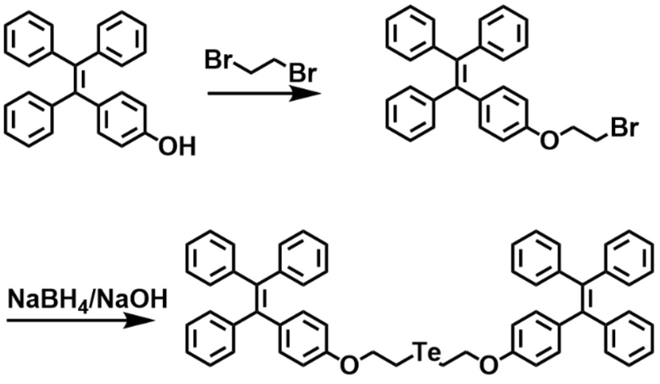

Scheme S1. Synthetic routes of $(TPE\text{-}C2)_2$-Te.

Synthesis of TPE-C_2-Br

4-(1,2,2-triphenylvinyl) phenol (0.2 g, 0.57 mmol) was dissolved in 50 mL of acetonitrile, K_2CO_3 (0.6 g, 4.3 mmol) and 1,2-dibromoethane (0.8 g, 4.3 mmol) were added. The mixture was refluxed for 4 h before cooling and filtering. Then solvent was removed under vacuum, the residue re-dissolved in CH_2Cl_2 (30 mL). The filtrate was concentrated and purified on a silica gel column with an eluent of petroleum ether and DCM (1:1) to give compound TPE-C_2-Br as yellow solid (0.11 g).

^{1}H NMR (400MHz, $CDCl_3$): δ =7.12-7.03 (m, 15H), 6.95-6.93 (d, 2H), 6.65-6.63 (d, 2H), 4.23-4.20 (t, 2H), 3.61-3.58 (t, 2H) ppm.

^{13}C NMR (100MHz, $CDCl_3$): δ =156.57, 143.86, 140.32, 136.92, 132.62, 131.35, 131.32, 127.73, 127.62, 126.40, 126.28, 113.84, 67.68, 29.10 ppm.

ESI-MS ($C_{28}H_{23}BrO$) m/z: calcd. for $[M]^+$ 454.093; found 454.093.

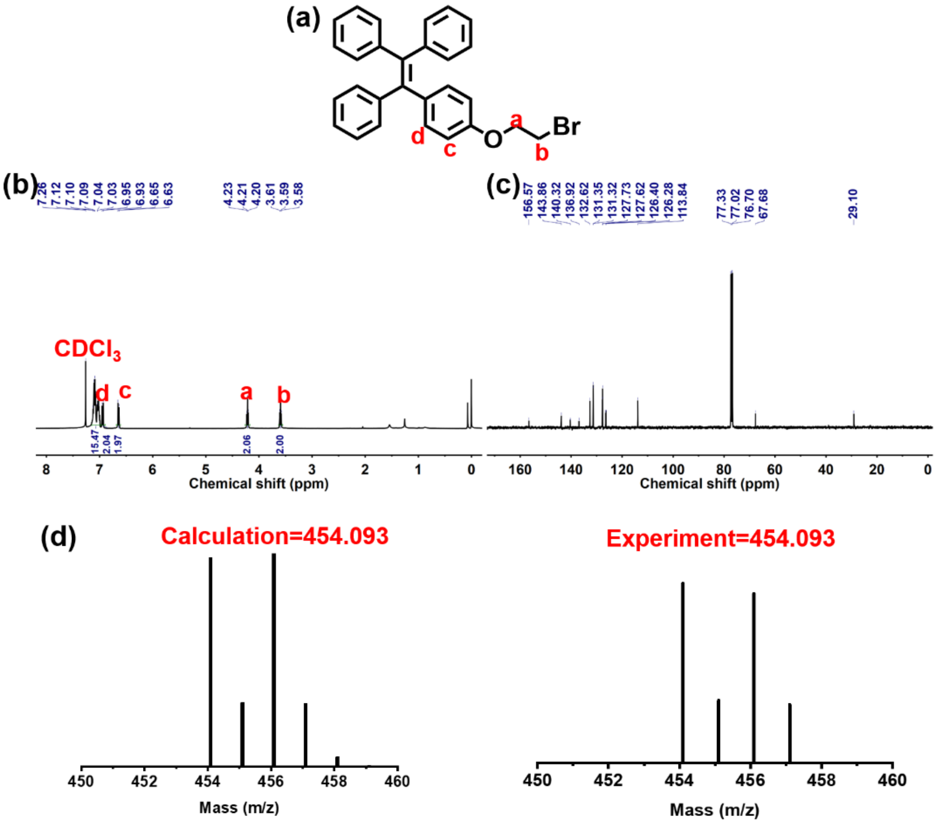

Figure S1. Characterization of TPE-C_2-Br: (a) Structural formula; (b) ^{1}H-NMR spectrum; (c) ^{13}C-NMR spectrum and (d) ESI-MS spectrum.

Synthesis of $(TPE\text{-}C2)_2$-Te

A total tellurium powder (0.06g, 0.46 mmol) and sodium borohydride (0.044g, 1.2 mmol) were added to 5 mL of water under the atmosphere of nitrogen. After 30 min of stirring under at 50 °C, TPE-C_2-Br (0.12 g, 0.27 mmol) dissolved in THF (5 mL) was added. The reaction was stirred at 50 °C for 12 h. Then solvent was removed under vacuum, the residue re-dissolved in CH_2Cl_2 (30 mL). The combined organic phase was concentrated and purified by flash column chromatography to afford compound $(TPE\text{-}C2)_2$-Te as yellow solid (0.032 g).

^{1}H NMR (400MHz, $CDCl_3$): δ =7.10-7.02 (m, 30H), 6.93-6.90 (d, 4H), 6.62-6.60 (d, 4H), 4.27-4.23 (t, 4H), 3.01-2.98 (t, 4H) ppm.

^{13}C NMR (100MHz, $CDCl_3$): δ =156.82, 143.93, 140.45, 140.19, 136.47, 132.58, 131.37, 127.72, 127.60, 126.37, 126.27, 113.77, 69.90, 1.81 ppm.

ESI-MS ($C_{56}H_{46}O_2Te$) m/z: calcd. for $[M+K]^+$ 919.220; found 919.219.

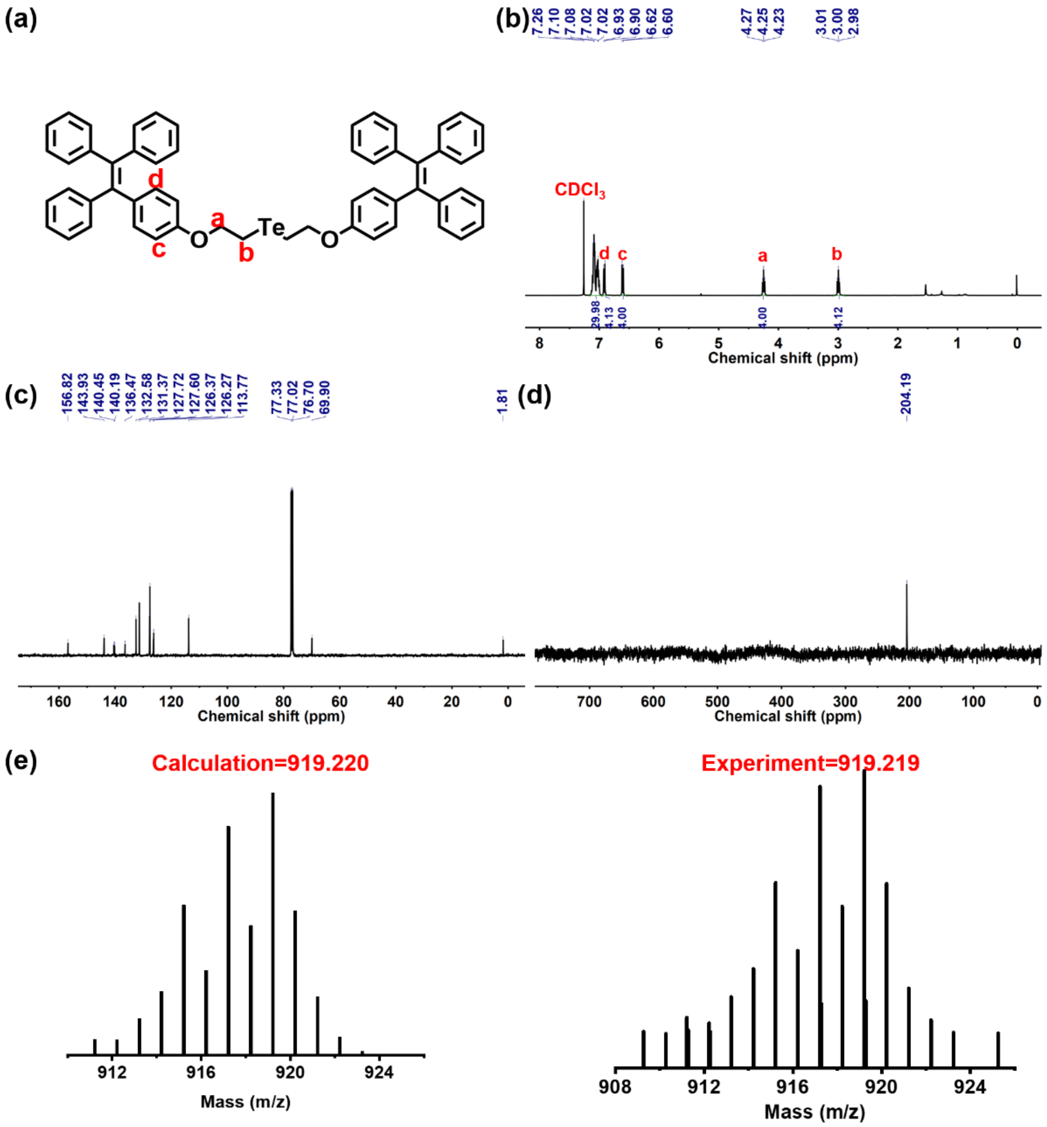

Figure S2. Characterization of $(TPE\text{-}C2)_2$-Te: (a) Structural formula; (b) ^{1}H-NMR spectrum; (c) ^{13}C-NMR spectrum; (d) ^{125}Te-NMR spectrum and (e) ESI-MS spectrum.

2.2 Synthesis of $(TPE\text{-}C6)_2$-Te

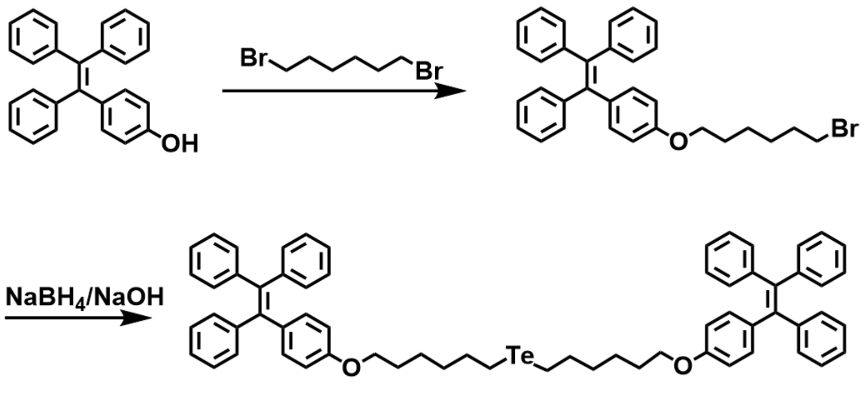

Scheme S2. Synthetic routes of $(TPE\text{-}C6)_2$-Te.

Synthesis of TPE-C_6-Br[1]

4-(1,2,2-triphenylvinyl) phenol (0.2 g, 0.57 mmol) was dissolved in 50 mL of acetonitrile, K_2CO_3 (0.6 g, 4.3 mmol) and 1,6-dibromohexane (1.03 g, 4.3 mmol) were added. The mixture was refluxed for 4 h before cooling and filtering. Then solvent was removed under vacuum, the residue re-dissolved in CH_2Cl_2 (30 mL). The filtrate was concentrated and purified on a silica gel column with an eluent of petroleum ether and DCM (1:1) to give compound TPE-C_6-Br as yellow solid (0.05 g).

^{1}H NMR (400MHz, $CDCl_3$): δ =7.10-7.00 (m, 15H), 6.93-6.91 (d, 2H), 6.63-6.61 (d, 2H), 3.90-3.86 (t, 2H), 3.44-3.40 (t, 2H), 1.92-1.85 (m, 2H), 1.77-1.74 (m, 2H), 1.54-1.47 (m, 4H) ppm.

^{13}C NMR (100MHz, $CDCl_3$): δ =157.62, 144.04, 140.61, 140.06, 136.03, 132.56, 131.37, 127.63, 126.25, 113.61, 33.83, 32.72, 29.17, 27.99, 25.37 ppm.

ESI-MS ($C_{32}H_{31}BrO$) m/z: calcd. for $[M+H]^+$ 551.118; found 551.118.

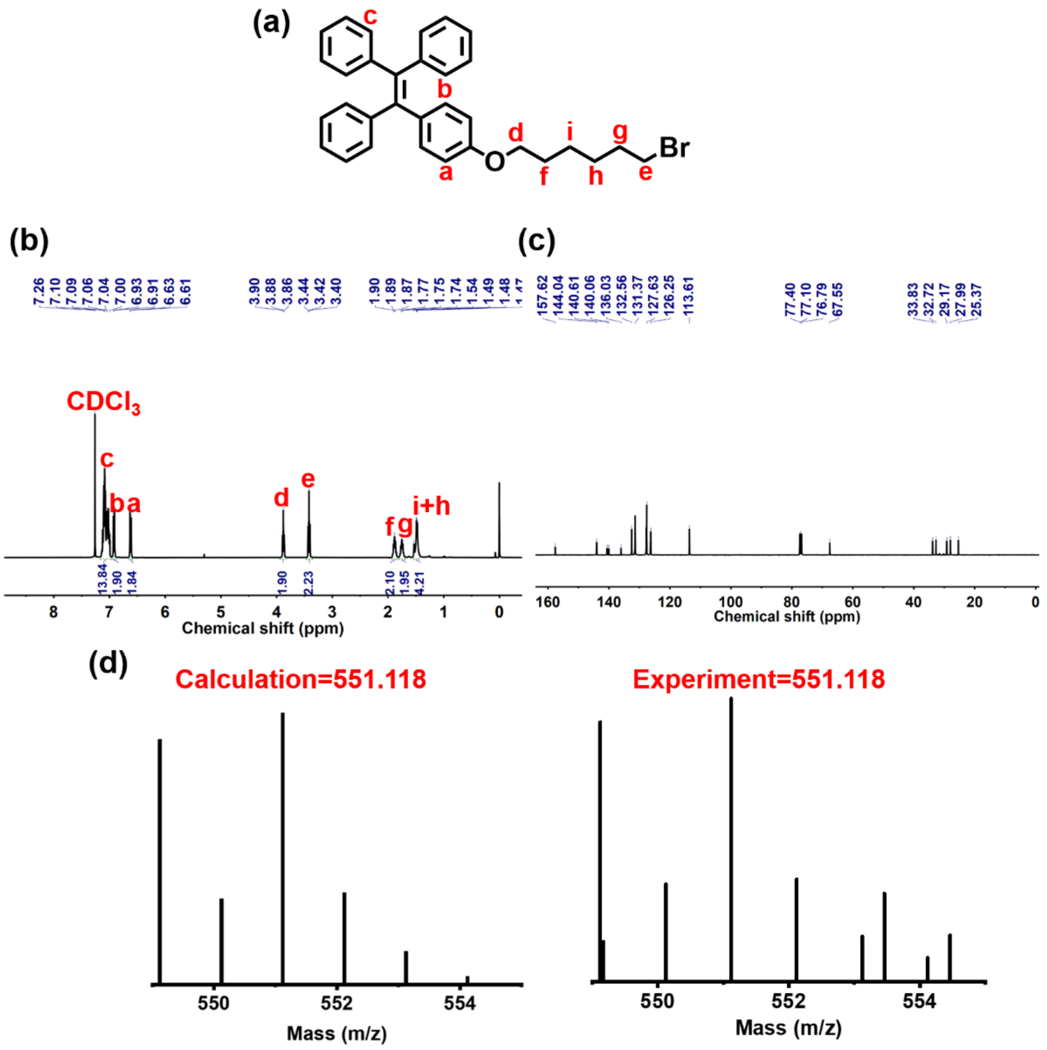

Figure S3. Characterization of TPE-C_6-Br: (a) Structural formula; (b) ^{1}H-NMR spectrum; (c) ^{13}C-NMR spectrum and (d) ESI-MS spectrum.

Synthesis of (TPE-C6)$_2$-Te

A total tellurium powder (0.06g, 0.46 mmol) and sodium borohydride (0.044g, 1.2 mmol) were added to 5 mL of water under the atmosphere of nitrogen. After 30 min of stirring under at 50 °C, TPE-C_6-Br (0.13 g, 0.25 mmol) dissolved in THF (5 mL) was added. The reaction was stirred at 50 °C for 12 h. Then solvent was removed under vacuum, the residue re-dissolved in CH_2Cl_2 (30 mL). The combined organic phase was concentrated and purified by flash column chromatography to afford compound (TPE-C6)$_2$-Te as yellow solid (0.042 g).

^{1}H NMR (400MHz, $CDCl_3$): δ =7.11-7.03 (m, 30H), 6.94-6.92 (d, 4H), 6.64-6.62 (d, 4H), 3.89-3.86

(t, 4H), 2.66-2.62 (t, 4H), 1.81-1.71 (m, 8H), 1.53-1.44 (m, 8H) ppm.

^{13}C NMR (100MHz, $CDCl_3$): δ =157.64, 144.07, 140.59, 140.01, 135.94, 132.51, 131.34, 127.71, 126.21, 113.58, 67.66, 32.18, 31.80, 29.22, 25.48, 1.82 ppm.

ESI-MS ($C_{64}H_{62}O_2Te$) m/z: calcd. for $[M+K]^+$ 1031.346; found 1031.345.

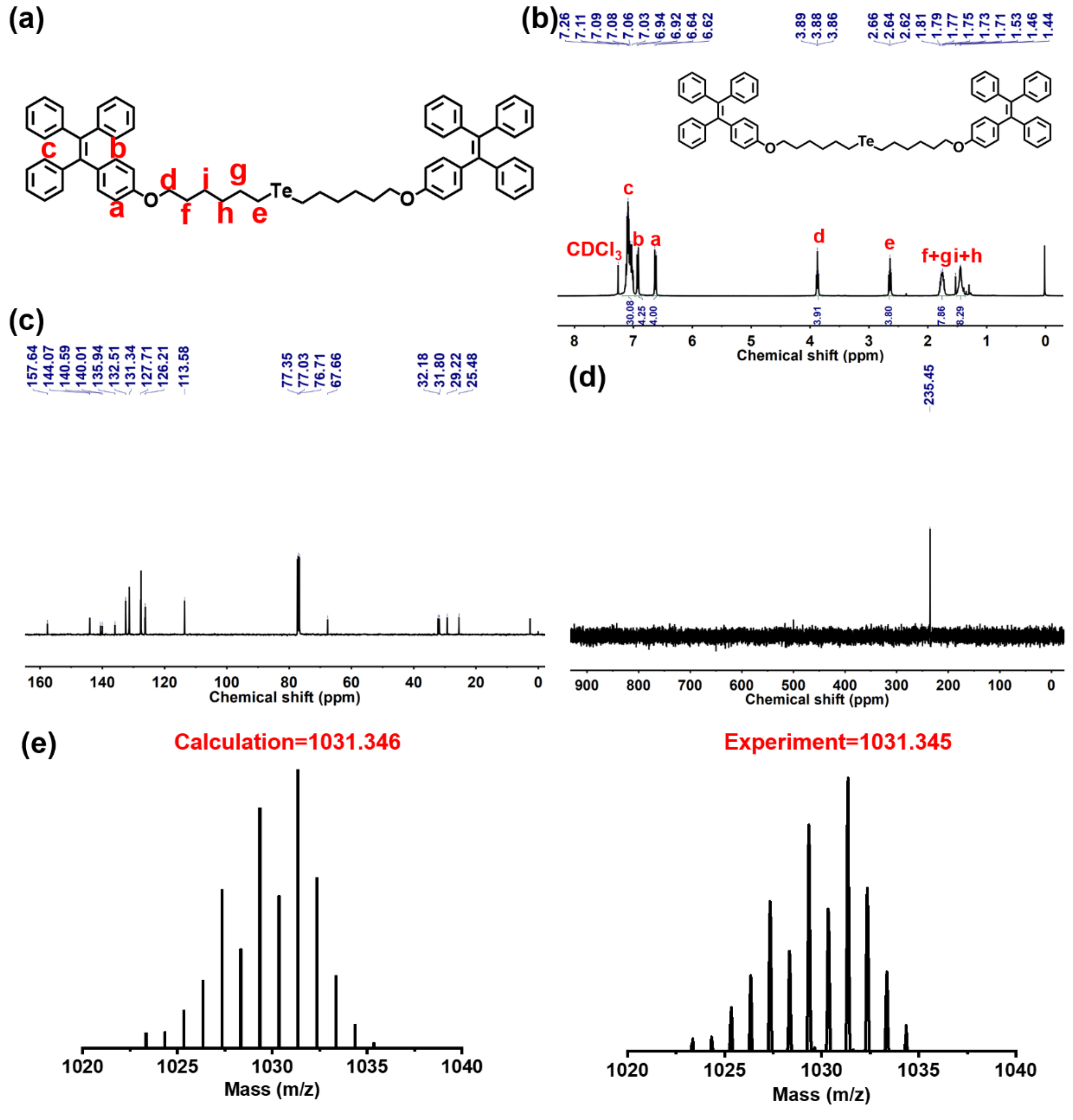

Figure S4. Characterization of (TPE-C6)$_2$-Te: (a) Structural formula; (b) ^{1}H-NMR spectrum; (c) ^{13}C-NMR spectrum; (d) ^{125}Te-NMR spectrum and (e) ESI-MS spectrum.

2.3 Synthesis of $(TPE\text{-}C12)_2$-Te

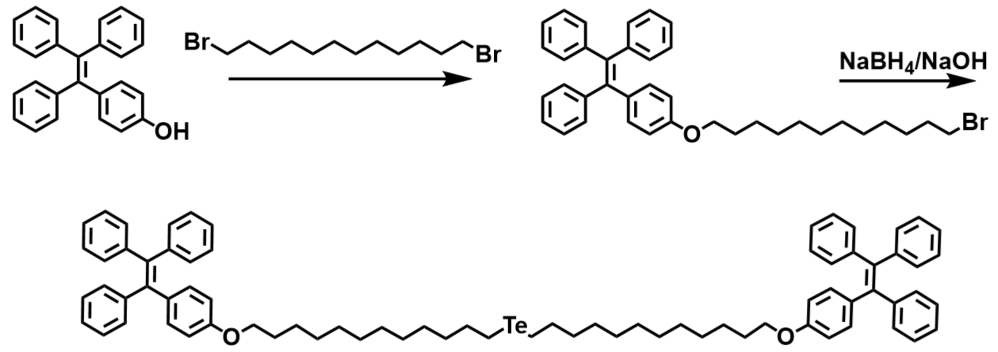

Scheme S3. Synthetic routes of $(TPE\text{-}C12)_2$-Te.

Synthesis of TPE-C_{12}-Br

4-(1,2,2-triphenylvinyl) phenol (0.2 g, 0.57 mmol) was dissolved in 50 mL of acetonitrile, K_2CO_3 (0.6 g, 4.3 mmol) and 1,12-dibromododecane (1.41 g, 4.3 mmol) were added. The mixture was refluxed for 4 h before cooling and filtering. Then solvent was removed under vacuum, the residue re-dissolved in CH_2Cl_2 (30 mL). The filtrate was concentrated and purified on a silica gel column with an eluent of petroleum ether and DCM (1:1 v/v) to give compound TPE-C_{12}-Br as yellow solid (0.08 g).

^{1}H NMR (400MHz, DMSO-d_6): δ =7.12-6.99 (m, 15H), 6.93-6.90 (d, 2H), 6.63-6.61 (d, 2H), 3.89-3.85 (t, 2H), 3.43-3.39 (t, 2H), 1.89-1.82 (m, 2H), 1.75-1.69 (m, 2H), 1.42-1.48 (m, 16H) ppm.

^{13}C NMR (100MHz, DMSO-d_6): δ =157.70, 144.04, 140.61, 139.98, 135.88, 132.49, 131.40, 127.70, 127.57, 126.32, 126.19, 113.57, 67.82, 53.43, 34.05, 32.86, 29.54, 29.44, 29.32, 28.78, 28.19, 26.07 ppm.

ESI-MS ($C_{38}H_{43}OBr$) m/z: calcd. for $[M+K]^+$ 635.212; found 635.212.

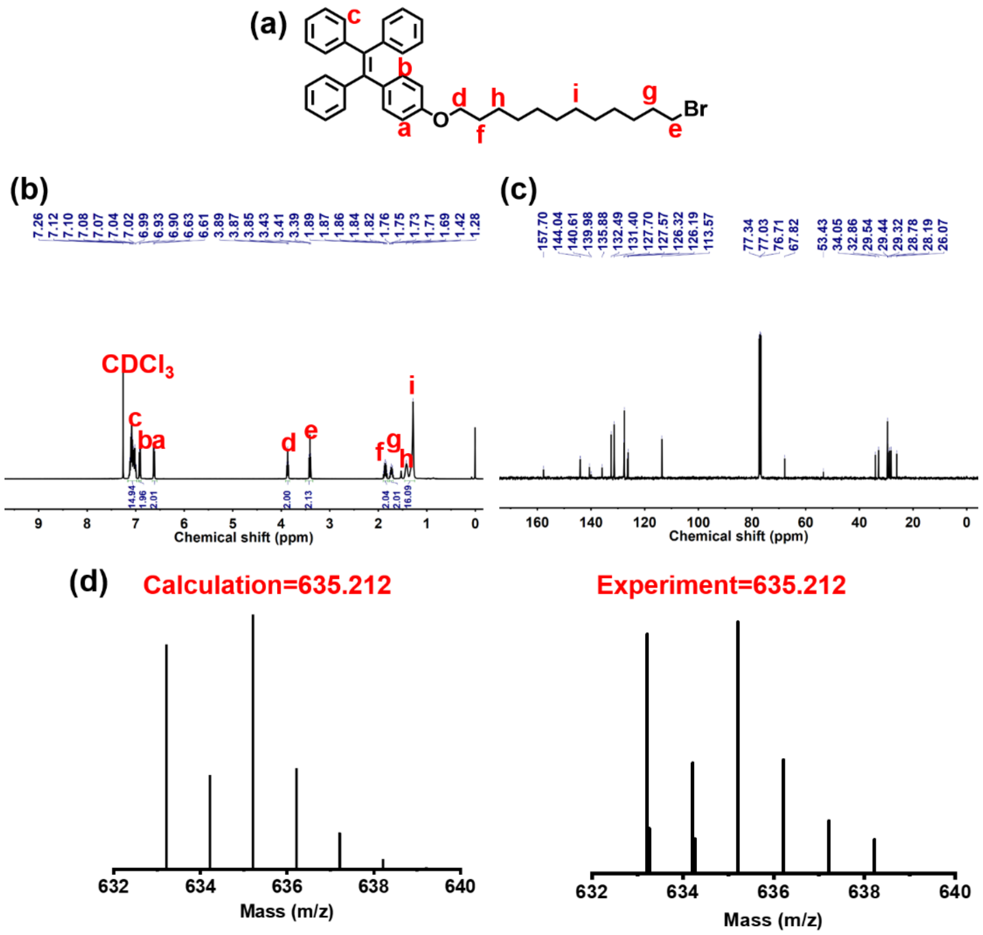

Figure S5. Characterization of TPE-C_{12}-Br: (a) ^{1}H-NMR spectrum; (b) ^{13}C-NMR spectrum and (c) ESI-MS spectrum.

Synthesis of (TPE-C12)$_2$-Te

A total tellurium powder (0.06g, 0.46 mmol) and sodium borohydride (0.044g, 1.2 mmol) were added to 5 mL of water under the atmosphere of nitrogen. After 30 min of stirring under at 50 °C, TPE-C_{12}-Br (0.16 g, 0.27 mmol) dissolved in THF (5 mL) was added. The reaction was stirred at 50 °C for 12 h. Then solvent was removed under vacuum, the residue re-dissolved in CH_2Cl_2 (30 mL). The combined organic phase was concentrated and purified by flash column chromatography to afford compound (TPE-C12)$_2$-Te as yellow solid (0.02 g).

^{1}H NMR (400MHz, $CDCl_3$): δ =7.12-7.01 (m, 30H), 6.93-6.91 (d, 4H), 6.64-6.62 (d, 4H), 3.89-3.86 (t, 4H), 2.65-2.61 (t, 4H), 1.76-1.70 (m, 8H), 1.34-1.28 (m, 34H) ppm.

^{13}C NMR (100MHz, $CDCl_3$): δ =157.71, 144.03, 140.62, 139.98, 135.87, 132.48, 131.39, 127.68, 127.56, 126.18, 113.59, 67.84, 32.30, 32.07, 29.56, 29.42, 29.32, 28.99, 26.07, 1.85 ppm.

ESI-MS ($C_{76}H_{86}O_2Te$) m/z: calcd. for $[M+K]^+$ 1199.533; found 1199.532.

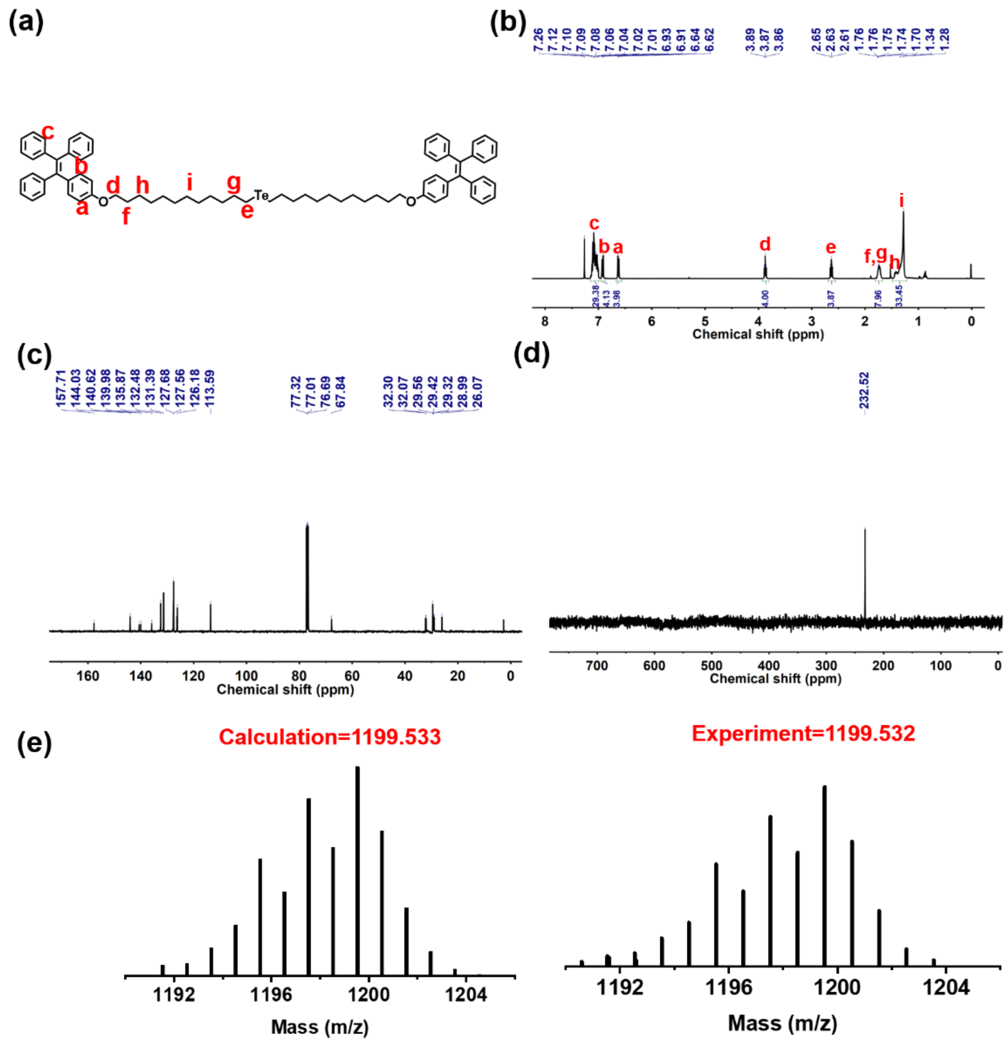

Figure S6. Characterization of $(TPE\text{-}C12)_2\text{-}Te$: (a) Structural formula; (b) ^{1}H-NMR spectrum; (c) ^{13}C-NMR spectrum; (d) ^{125}Te-NMR spectrum and (e) ESI-MS spectrum.

3. Results and discussion

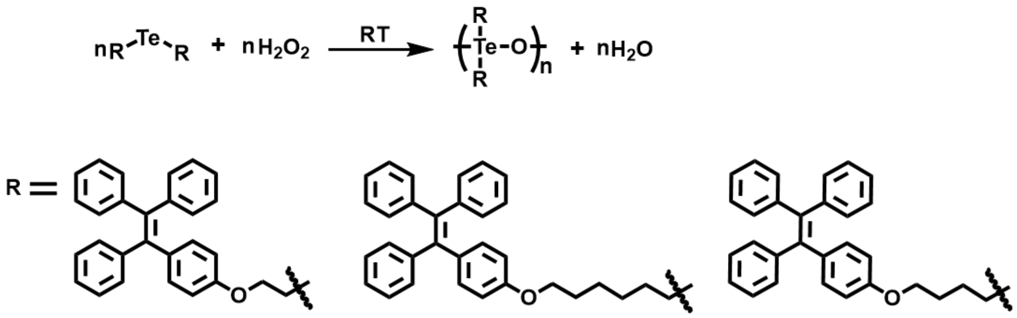

Figure S7. The formula of oxidation polymerization.

3.1 Relationship between polymer chain growth and fluorescence properties

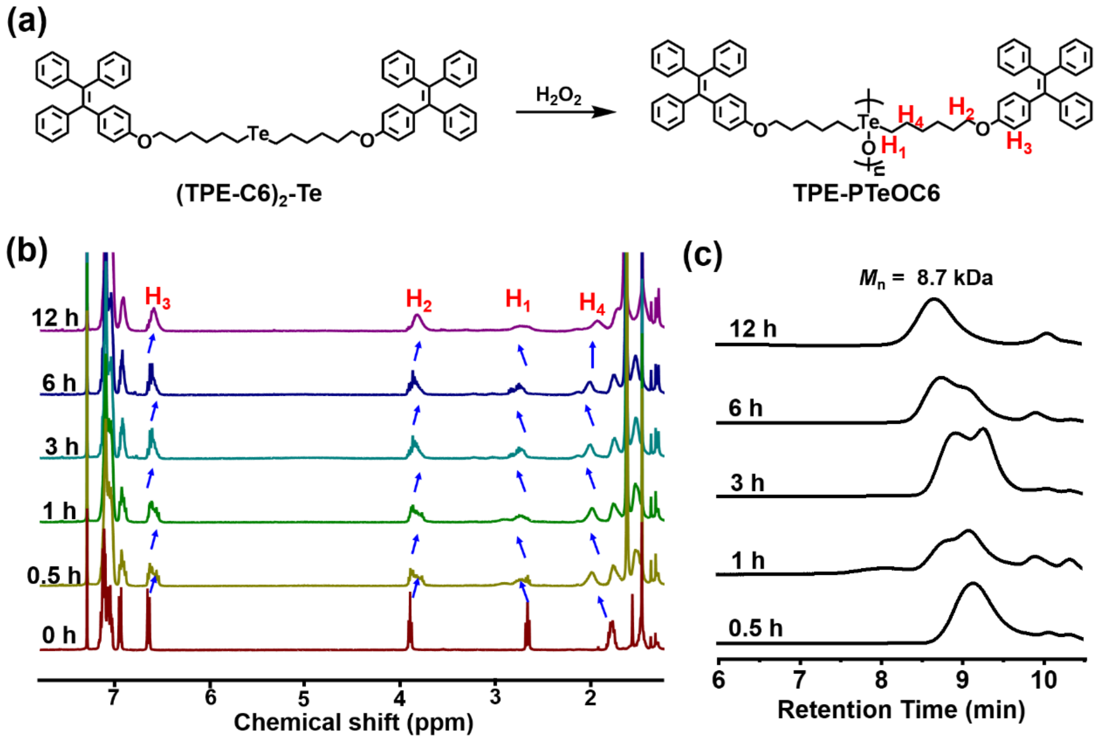

Figure S8. Confirmation of the formation of TPE-PTeOC6. (a) The polymerization process of TPE-PTeOC6. (b) ^{1}H NMR characterization of TPE-PTeOC6 obtained from (TPE-C6)$_2$-Te incubated with H_2O_2 for 12 h. (b) GPC analysis of the TPE-PTeOC6 obtained from (TPE-C6)$_2$-Te incubated with H_2O_2 at different time point, the result at 12 h giving a molecular weight of 0.87 kDa (PDI = 1.93).

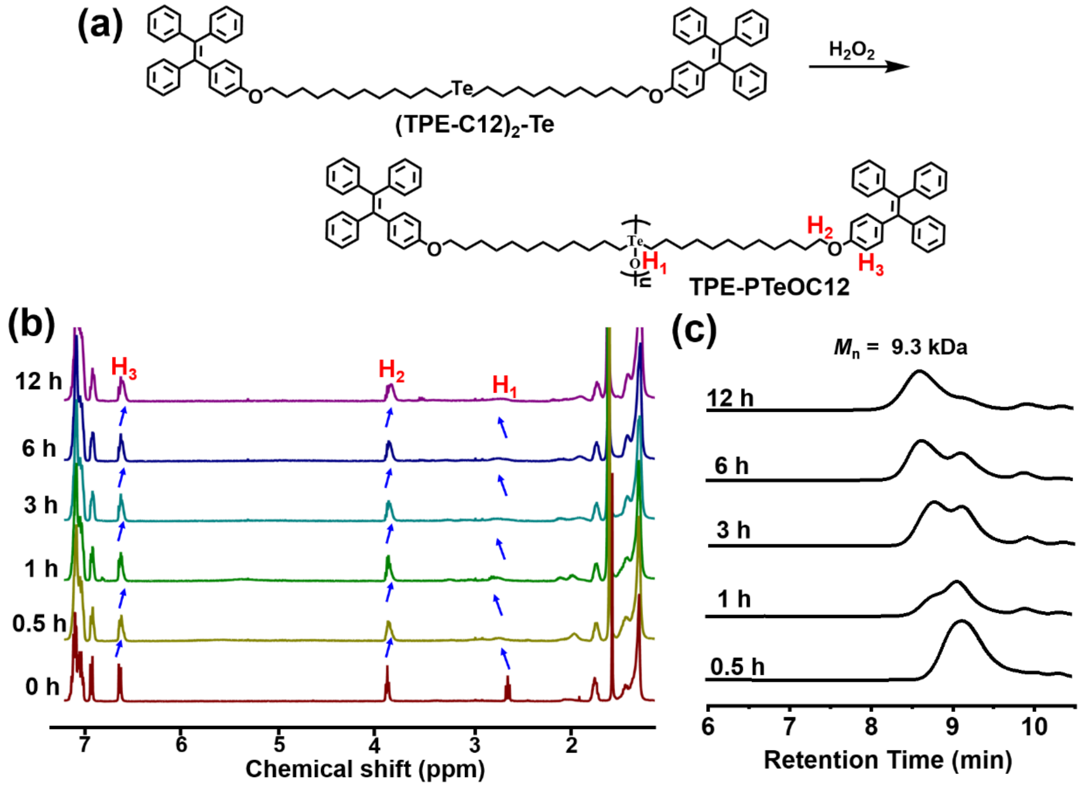

Figure S9. Confirmation of the formation of TPE-PTeOC12. (a) The polymerization process of TPE-PTeOC12. (b) ^{1}H NMR characterization of TPE-PTeOC12 obtained from (TPE-C12)$_2$-Te incubated with H_2O_2 for 12 h. (b) GPC analysis of the TPE-PTeOC12 obtained from (TPE-C12)$_2$-Te incubated with H_2O_2 at different time point, the result at 12 h giving a molecular weight of 0.93 kDa (PDI = 1.64).

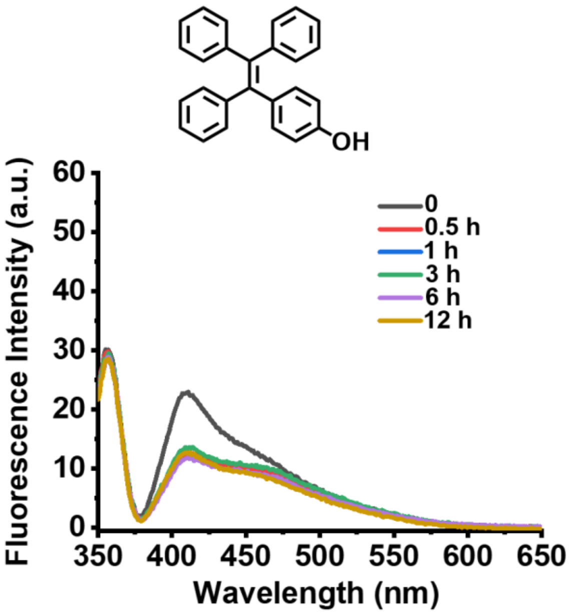

Figure S10. Fluorescent spectra of TPE treated with H_2O_2 at different time points.

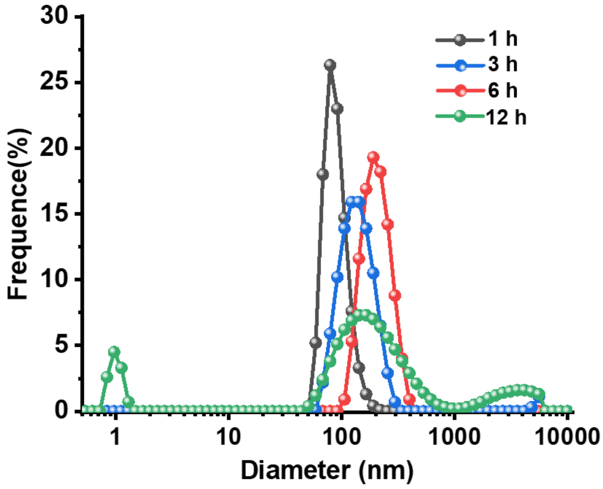

Figure S11. DLS of $(TPE-C2)_2$-Te after 1 h, 3 h, 6 h and 12 h incubation in the H_2O_2 solution.

3.2 Oxidative polymerization in surface

3.2.1 Synthesis of di-(valeric acid) tellurium $(HOOCC_4)_2Te$

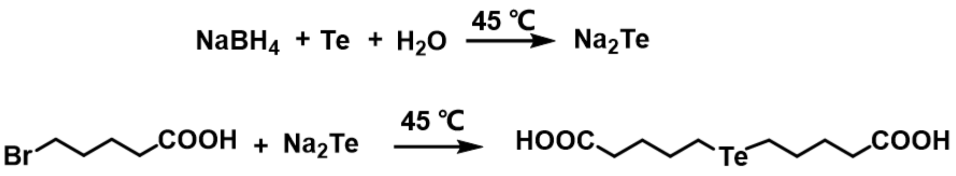

Scheme S4. Synthetic routes of $(HOOCC_4)_2Te$.

A total tellurium powder (0.03g, 0.23 mmol) and sodium borohydride (0.022g, 0.6 mmol) were added to 5 mL of water under the atmosphere of nitrogen. After 30 min of stirring under at 45 °C, 5-Bromovaleric acid (0.02 g, 0.12 mmol) dissolved in THF (5 mL) was added. The reaction was stirred at 45 °C for 12 h. Then solvent was removed under vacuum, the residue re-dissolved in CH_2Cl_2 (30 mL). Products were purified by filtration and recrystallization.

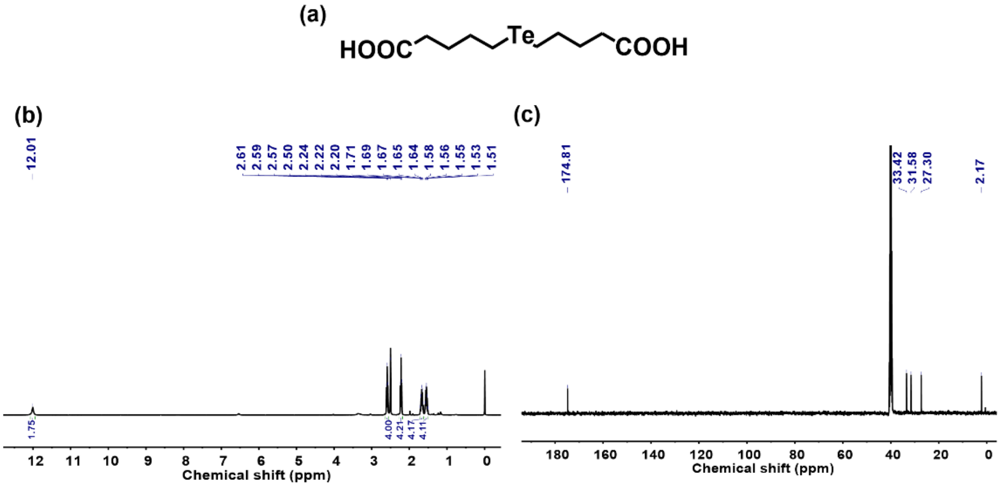

Figure S12. Characterization of $(HOOCC_4)_2Te$: ^{1}H-NMR spectrum and ^{13}C-NMR spectrum.

3.2.2 Preparation of tellurium-containing quartz surface (SiO_2-NHCOTe)

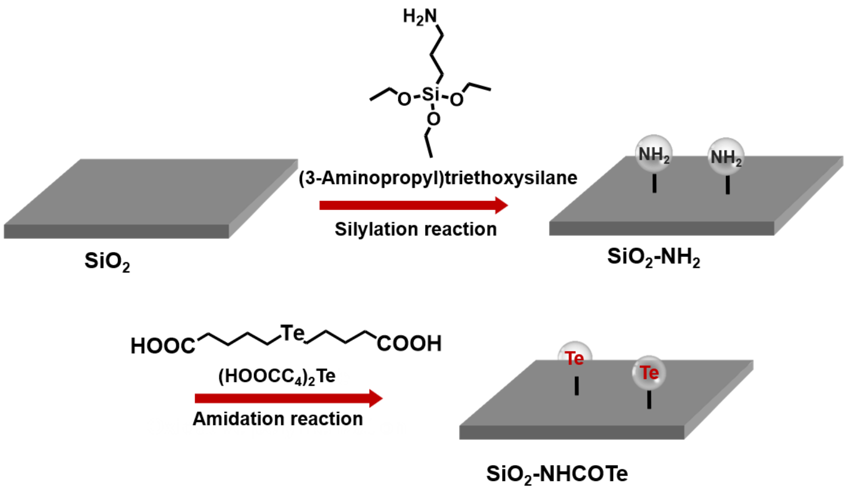

Figure S13. Procedure of modifying of SiO_2-NHCOTe.

According to our previous work,[2] the quartz substrate was first modified with 3-aminopropyltriethoxysilane in toluene by a silylation reaction. Then, the modified surface with amino groups was then treated with $(HOOCC_4)_2Te$ to obtain the tellurium-containing quartz surface denoted as SiO_2-NHCOTe. For a standard surface modification process, 0.2 mol/L $(HOOCC_4)_2Te$ was dissolved in chloroform and was then covered on the SiO_2-NH_2 surface. The obtained surface was washed with acetone and dried by N_2 flow.

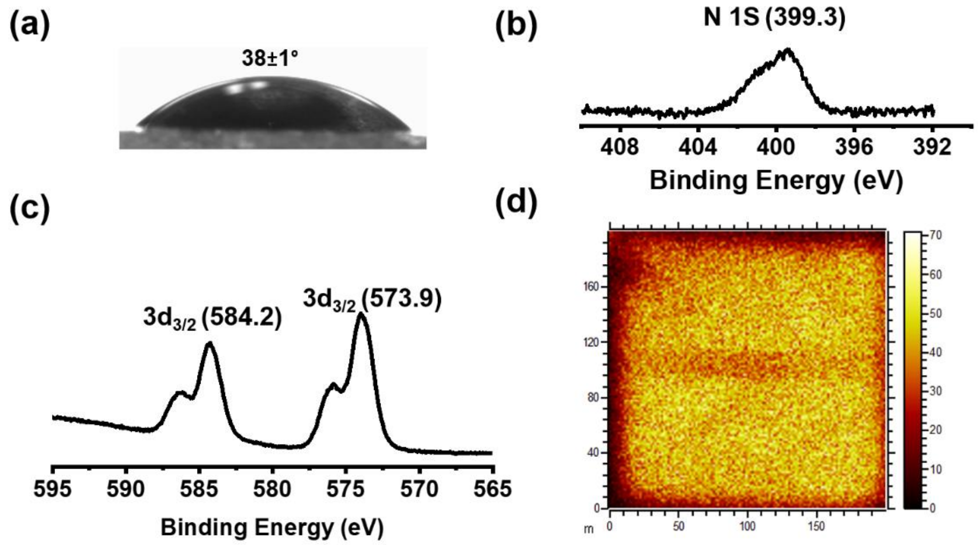

Figure S14. Characterization of SiO_2-NHCOTe. (a) Static water contact angle of SiO_2-NH_2. (b) XPS N 1s spectrum of the surface SiO_2-NH_2. (c) XPS Te 3d spectrum of the surface SiO_2-NHCOTe. (d) ToF-SIMS map of Te^- ions on the SiO_2-NHCOTe surface.

To confirm the successful modification of amino functional groups, a static water contact angle (WAC) experiment was first conducted. For the unmodified SiO_2-NH_2 surface, WAC was 38±1°C (Figure S12a). After silylation reaction, the WAC increased to 53±2°C as a result of the relatively hydrophobic aliphatic structure. The single peak for N 1s spectra shown in the XPS of SiO_2-NH_2 surface (Figure S12b), verifying the successful modification of amino functional groups. Then, SiO_2-NHCOTe surface was prepared through amidation reaction between SiO_2-NH_2 and $(HOOCC_4)_2Te$. In Figure S12c, the binding energies of Te at 584.2 and 573.9 eV were attributed to Te 3d, verifying the chemical structure of the tellurium. Additionally, we also used time-of-flight secondary ion mass spectrometry (ToF-SIMS) to scan the tellurium surface. It can be seen that for a 200 X 200 μm area, Te was uniformly distributed on the surface (Figure S12d). All these results indicated SiO_2-NHCOTe was successfully constructed.

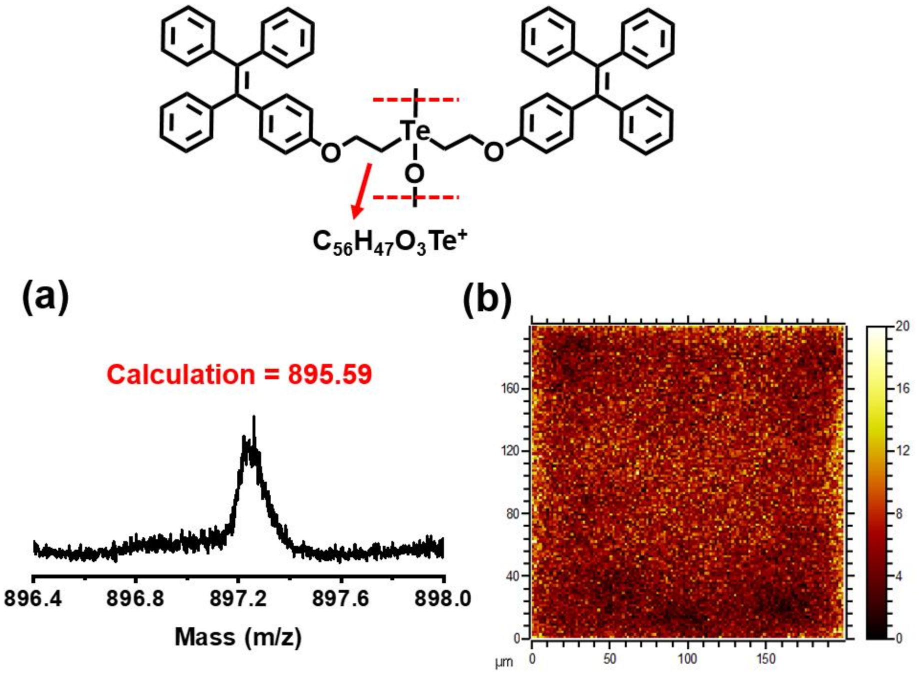

Figure S15. (a) Observed ToF-SIMS result of the fragment, *M* 895.59. (b) ToF-SIMS map of $[M+H]^+$($C_{56}H_{47}O_3Te^+$) ions on the SiO_2-TPE-PTeOC2 surface.

3.3 Acid-Responsiveness of TPE-PTeOC2.

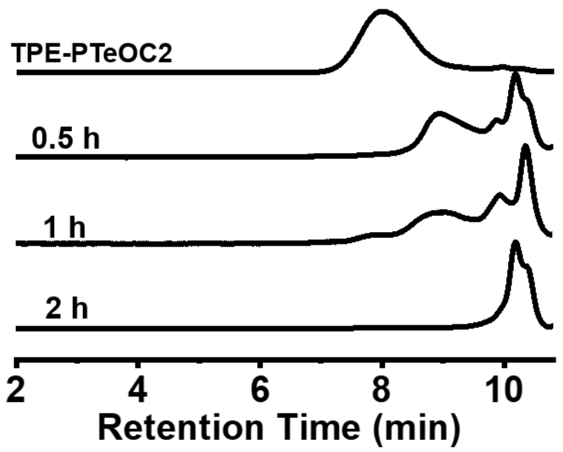

Figure S16. GPC analysis of the TPE-PTeOC2 incubated with 0.5 mM HCl for 0.5, 1, and 2 h.

4. References

[1] N. Zhang, H. Chen, Y. Fan, L. Zhou, S. Trepout, J. Guo, M. Li. *ACS Nano* **2018**, *12*, 4025-4035.

[2] J. Xia, P. Zhao, K. Zheng, C. Lu, S. Yin, H. Xu. *Angew. Chem. Int. Ed.* **2019**, *58*, 542-546.
